# Snail-Inspired Metabolic Rewiring Offers a New Avenue to Cardioprotection

**DOI:** 10.20517/jca.2026.101

**Published:** 2026-08-30

**Authors:** Xuejun Wang, Neha Dagar

**Affiliations:** Division of Biomedical and Translational Sciences, University of South Dakota Sanford School of Medicine, Vermillion, South Dakota, USA.

## Abstract

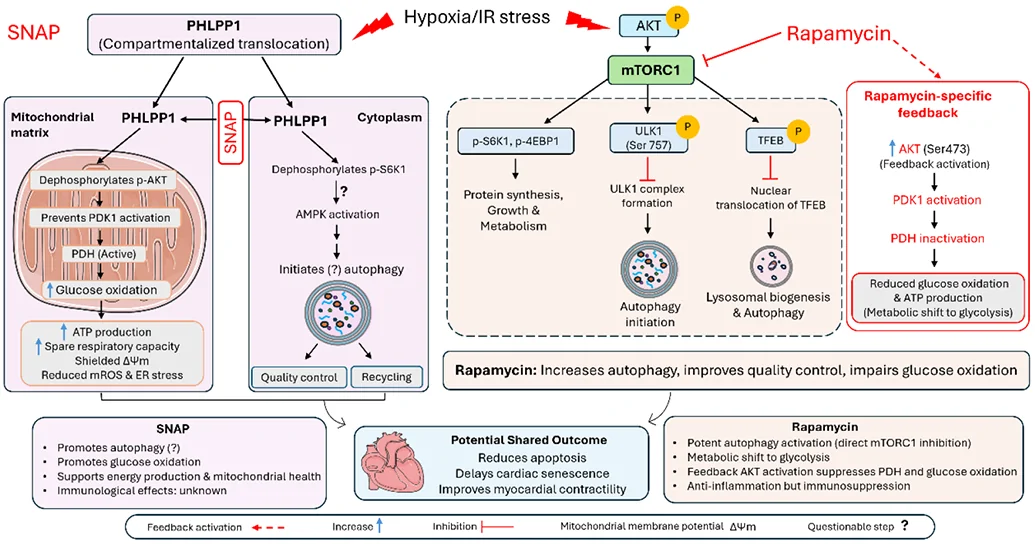

## BRIDGING THE EVOLUTIONARY DIVIDE

A primary challenge in cardiovascular medicine and aging research is developing robust therapies to shield the myocardium from ischemia-reperfusion (IR) injury. In humans and non-hibernating model organisms, acute ischemia initiates a destructive cascade marked by rapid mitochondrial depolarization, runaway reactive oxygen species (mROS) generation, ER stress, and cell death ^[1, 2]^. Conversely, natural hibernators and aestivators cross these severe metabolic valleys unharmed. These species depress their heart rate, respiratory capacity, and core temperature for months, returning to full physiological function without structural or functional impairment ^[3]^.

In a recently published study^[4]^, Piao et al. bridge this evolutionary chasm. They isolated and characterized a circulating dormancy-inducing factor (DIF) of aestivating land snails (*Oreohelix subrudis*). Chemically synthesizing this DIF yielded a first-in-class, small-molecule allosteric activator of PH domain and leucine-rich repeat protein phosphatase 1 (PHLPP1), which they named **SNAP** (**SN**ail **A**ctivator of **P**HLPP1) after its precise molecular target. Treating non-hibernating mammalian cells and perfused mouse hearts with SNAP artificially engineers a protective, "hibernation-like" state. This chemical intervention provides profound structural and functional protection against IR stress. This discovery carries profound implications for the field of cardiovascular aging, where mitochondrial decline, compromised autophagic clearance, and heightened susceptibility to IR stress are among the hallmarks^[5, 6]^.

## STRESS-DEPENDENT COMPARTMENTALIZED METABOLIC REWIRING

SNAP selectively activates PHLPP1, a key phosphatase that modulates the pivotal AKT and mTORC1/S6K1 signaling networks. Under basal homeostatic conditions, these pathways dictate cell growth, proliferation, and metabolic rate. However, during the acute crisis of an IR insult, Piao et al. observed a remarkable subcellular phenomenon: both plasma membrane-bound PHLPP1 and phosphorylated AKT (p-AKT) translocate into the cytoplasm and the mitochondria. By administering SNAP, PHLPP1 effectively dephosphorylates mitochondrial p-AKT and cytoplasmic p-S6K1. This specific, targeted biochemical rewiring induces a transient, reversible state of cellular dormancy, effectively shielding the cell from self-destruction during IR.

When mouse fibroblasts were subjected to severe IR stress, SNAP treatment triggered seemingly autophagy, preserved cellular proteostasis, and rendered the cells resistant to apoptosis. Crucially, rather than driving cells into irreversible senescent states, a common failure mechanism in the aging heart, SNAP prompted a clean, reversible cell-cycle exit. In further testing using isolated adult mouse cardiomyocytes and perfused whole hearts, SNAP provided profound, holistic cardioprotection. It sustained mitochondrial membrane potential, reduced mROS, and crucially preserved Pyruvate Dehydrogenase (PDH) activity. The authors elegantly confirmed this precise mitochondrial mechanism of protection by demonstrating that SNAP's beneficial effects were abolished in mouse hearts with cardiomyocyte-specific PDH knockout.

Concurrently, in the cytoplasm, SNAP-driven PHLPP1 dephosphorylates and inhibits p-S6K1, a downstream effector of mTORC1, where the reduction of p-S6K is associated with increases in p-Thr172-AMPK and LC3B-II, a marker of autophagic vacuoles. Notably, evidence provided by the authors is consistent with a potential mediating role of autophagy activation in SNAP elicited protection but is far from definitive. This is because autophagic flux was not rigorously assessed and the necessity of autophagic activation in SNAP’s cardioprotection remains to be determined. Mechanistically, the authors emphasize the PHLPP1-S6K1 axis without the direct involvement of mTORC1 or AKT in SNAP-induction of autophagy. To this end, it will be important to address how SNAP impacts TFEB activation and thereby lysosomal genesis^[7, 8]^.

## EFFECTS OF SNAP ON SIMULATED CARDIAC IR INJURY

Just as SNAP treatment produces no discernible effects in cultured fibroblasts and cardiomyocytes under normal culture conditions, pretreating ex vivo perfused hearts with SNAP during the non-ischemic phase (PO2 100mmHg, glucose 10mM) preceding simulated global ischemia yielded no significant effects on subsequent ischemia (30 min) and reperfusion (40 min) regarding left ventricular (LV) functional recovery and arrhythmia ^[4]^. However, SNAP treatment during the moderate ischemic phase (PO2 50mmHg, glucose 5mM; 30 min) immediately preceding the complete global ischemia phase and instigated to mimic the preservation phase of donor hearts for transplantation provided significant protection against subsequent IR injury, as detected at 10, 20, and 40 min after the initiation of reperfusion. The LV functional recovery of the SNAP-treated group was significantly better than that of the control group. Moreover, the reperfusion-phase LV functional parameters of the SNAP-treated group, but not the control group, seemed to rise above baseline values ^[4]^. Although these data show strikingly large variations and lack a longitudinal comparison between the reperfusion phase and the pre-global ischemia phases, they suggest that SNAP pretreatment not only preserves LV function but may also exert a delayed stimulating effect. Notably, in the Langendorff ex vivo cardiac IR model, all but one parameter (the time to return to stable contraction during reperfusion) did not show protective effects from SNAP treatment when the global ischemia phase was extended to 40 min.

## SNAP VS. CALORIC RESTRICTION MIMETICS (CRMS)

The mechanism of SNAP introduces an intriguing contrast to established CRMs like rapamycin. Dampening nutrient-sensing pathways using CRMs have long been the gold standard in geroscience to delay cardiovascular aging and prolong lifespan. By directly inhibiting mTORC1^[6]^, Rapamycin operates via broad, systemic nutrient-deprivation signaling, which often presents clinically restrictive trade-offs, such as dysregulated glucose metabolism, impaired wound healing, and chronic immunosuppression^[9]^. In contrast, SNAP rewires metabolic signaling in a stress dependent and highly compartmentalized fashion. Instead of globally shutting down mTORC1 and AKT, SNAP selectively hijacks the localized stress response via PHLPP1 activation. This allows SNAP to target the mitochondrial fractions of p-AKT and cytoplasmic p-S6K1 concurrently. A notable advantage of SNAP is its preserving PDH activity and thereby glucose oxidation while upregulating autophagy, safeguarding mitochondrial polarization against acute mROS damage. This targeted activation pattern allows SNAP to trigger protective dormancy and likely autophagic activation locally within ischemic tissue, potentially avoiding the toxic systemic metabolic side effects that handicap lifelong rapamycin therapy^[9]^. Of course, the effect of systemically administered SNAP is currently unknown.

## CLINICAL TRANSLATION AND COMPLEXITIES

The stated advantage of SNAP compared with rapamycin is presently based exclusively on findings from an ex vivo IR injury model and cell cultures. Whether SNAP is suitable for chronic or even acute systemic administration remains to be tested, let alone whether it exerts the same protective effects as suggested by ex vivo and in vitro studies. However, translating this research into the arena of solid organ transplantation may be more immediately attainable. Currently, the preservation of donor hearts relies almost exclusively on hypothermia to suppress metabolic demand. Despite this cold storage, ischemic damage continues to accumulate, confining viable organ transit times to a narrow four-to-six-hour window and excluding many older or marginal donor organs from utilization. Incorporating a pharmacological dormancy inducer like SNAP directly into cardioplegic solutions could fundamentally revolutionize *ex vivo* organ preservation. By mimicking the molecular defense strategy of an estivating snail, clinicians could theoretically place donor hearts into a highly protected, "hibernated" state at room or low temperatures, safeguarding mitochondrial architecture, minimizing reperfusion-induced ROS cascades, and significantly extending the geographical and temporal boundaries of transplantation networks.

Transitioning SNAP from a preclinical proof-of-concept to human clinical trials will undoubtedly require overcoming substantial hurdles. Future studies must comprehensively evaluate SNAP’s safety profile, pharmacokinetics, and systemic clearance properties in large animal models. It is vital to confirm that the enforced cell-cycle arrest and metabolic suppression remain completely and safely reversible within complex, intact human organs without inducing long-term functional deficits. Additionally, understanding how SNAP interacts with pre-existing comorbid states typical of an aging patient population, such as chronic microvascular inflammation or severe atherosclerosis, will be essential to defining its therapeutic boundaries.

In conclusion, the study by Piao et al. underscores the immense, often untapped value of leveraging evolutionary adaptations to address severe human medical challenges. By carefully parsing the biochemical architecture of snail hibernation, the authors have introduced an exciting new tool for cardiac preservation and metabolic management. This pioneering strategy of inducing adaptive cellular dormancy not only adds a new approach to treating acute IR injuries but also shines profound insights into rescuing the fragile, aging cardiovascular system from the brink of metabolic failure.
